# ATF3, an HTLV-1 bZip factor binding protein, promotes proliferation of adult T-cell leukemia cells

**DOI:** 10.1186/1742-4690-8-19

**Published:** 2011-03-17

**Authors:** Keita Hagiya, Jun-ichirou Yasunaga, Yorifumi Satou, Koichi Ohshima, Masao Matsuoka

**Affiliations:** 1 Laboratory of Virus Control, Institute for Virus Research, Kyoto University, 53 Shogoin Kawahara-cho, Sakyo-ku, Kyoto 606-8507, Japan; 2 Department of Pathology, School of Medicine, Kurume University, 67 Asahimachi, Kurume, Fukuoka 830-0011, Japan

## Abstract

**Background:**

Adult T-cell leukemia (ATL) is an aggressive malignancy of CD4^+ ^T-cells caused by human T-cell leukemia virus type 1 (HTLV-1). The *HTLV-1 bZIP factor *(*HBZ*) gene, which is encoded by the minus strand of the viral genome, is expressed as an antisense transcript in all ATL cases. By using yeast two-hybrid screening, we identified activating transcription factor 3 (ATF3) as an HBZ-interacting protein. ATF3 has been reported to be expressed in ATL cells, but its biological significance is not known.

**Results:**

Immunoprecipitation analysis confirmed that ATF3 interacts with HBZ. Expression of ATF3 was upregulated in ATL cell lines and fresh ATL cases. Reporter assay revealed that ATF3 could interfere with the HTLV-1 Tax's transactivation of the 5' proviral long terminal repeat (LTR), doing so by affecting the ATF/CRE site, as well as HBZ. Suppressing ATF3 expression inhibited proliferation and strongly reduced the viability of ATL cells. As mechanisms of growth-promoting activity of ATF3, comparative expression profiling of ATF3 knockdown cells identified candidate genes that are critical for the cell cycle and cell death, including cell division cycle 2 (CDC2) and cyclin E2. ATF3 also enhanced p53 transcriptional activity, but this activity was suppressed by HBZ.

**Conclusions:**

Thus, ATF3 expression has positive and negative effects on the proliferation and survival of ATL cells. HBZ impedes its negative effects, leaving ATF3 to promote proliferation of ATL cells via mechanisms including upregulation of CDC2 and cyclin E2. Both HBZ and ATF3 suppress Tax expression, which enables infected cells to escape the host immune system.

## Background

Adult T-cell leukemia (ATL) is an aggressive CD4^+ ^T-cell malignancy caused by human T-cell leukemia virus type 1 (HTLV-1) [1-5]. In the plus strand of its genome, HTLV-1 encodes the regulatory proteins Tax and Rex and the accessory proteins p12, p30, and p13. The *HTLV-1 basic leucine zipper factor *(*HBZ*) gene is expressed as an antisense transcript. It has been reported that *HBZ *is consistently expressed and remains intact in all ATL cases and HTLV-1-infected individuals [6,7], where it promotes cell proliferation [6,8].

The *HBZ *gene is expressed as two isoforms: spliced HBZ (sHBZ) and unspliced HBZ (usHBZ) [9-12]. The expression of sHBZ in T-cells promotes T-cell proliferation whereas that of usHBZ does not [8,12]. HBZ was reported to repress Tax-mediated transactivation of viral transcription from the HTLV-1 promoter by dimerizing with transcription factors including cyclic AMP response element-binding protein 2 (CREB2), and members of the Jun family [10,13-16]. HBZ also promotes the degradation, directly and without ubiquitination, of some proteins that interact with HBZ [17]. Thus, HBZ interacts with host factors and modulates their function, which is likely to contribute to persistent infection of HTLV-1 *in vivo *and clonal expansion of infected cells.

Activating transcription factor 3 (ATF3) is a member of the ATF/cyclic AMP response element-binding (CRE) family of transcription factors [18]. *ATF3 *is an adaptive response gene whose expression is regulated by changes in the extra- or intracellular environment. ATF3 activates signals including DNA damage [19], anoxia [20], hypoxia [21], and represses others, including inflammation [22]. It can form homodimers or hetrodimers with other cellular bZIP transcription factors, including ATF2, c-Jun, JunB, and JunD, and exerts pleiotropic functions through ATF/CRE and AP-1 sites depending on cell type. It has also been pointed out that the *ATF3 *gene has a potential dichotomous role in cancer development [23]: it has pro-apoptotic functions, like a tumor suppressor, but at the same time induces cell proliferation, like an oncogene. It has been reported as up-regulated in malignant breast cancer cells [23], Hodgkin cells [24], and prostate cancer cells [25] where it is associated with proliferation. Transgenic mice overexpressing ATF3 in basal epithelial cells develop basal cell carcinomas [26]. Up-regulation of ATF3 is also reported in ATL cells [27], yet the biological significance in ATL is not known. Moreover, the question of how ATF3 induces proliferation of cancer cells remains unsolved.

In the process of elucidating the function of sHBZ in T-cells [6,12,28], we identified ATF3 as a sHBZ-interacting protein. In this study, we characterized the role of ATF3 in ATL cells. ATF3 was constitutively expressed in ATL cell lines and fresh ATL cases. ATF3 could repress Tax-mediated transactivation through ATF/CRE sites. Expression of ATF3 was linked to proliferation of ATL cells via upregulation of cell cycle-associated genes and down-regulation of proapoptotic genes. Furthermore, while ATF3 alone enhanced p53 stability, and therefore activation; sHBZ inhibited this function.

## Results

### Identification of ATF3 as a sHBZ interacting protein

**Figure 1:**
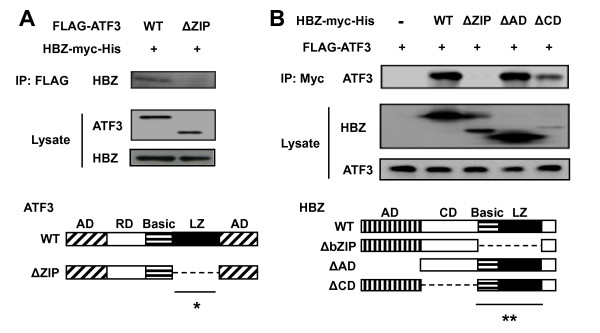
**Domains of ATF3 and sHBZ responsible for their interaction**. (A) Determination of the region of ATF3 responsible for the interaction with HBZ. 293FT cells were transfected with a FLAG-ATF3 mutant lacking the zipper domain along with sHBZ-Myc-His. 48 hours after transfection, total cell lysates were subjected to IP using anti-FLAG followed by IB using anti-His. (B) The region of HBZ responsible for interaction with ATF3. 293FT cells were transfected with the indicated tagged-HBZ mutants along with the FLAG-ATF3 vector. Cell lysates were subjected to IP using anti-Myc followed by IB using anti-FLAG. Schematic diagrams of ATF3 (A) and HBZ (B) are shown. AD, activation domain; RD, repression domain; LZ, leucine zipper; CD, central domain; WT, wild type. Asterisk (* or **) shows the region responsible for the molecular interaction.

We employed a yeast two-hybrid system with sHBZ as bait, to identify potential binding partners for sHBZ. Human activated mononuclear cell RP1 libraries were used for this screening and several candidates were identified (data not shown). Among them, we focused on ATF3 for the following reasons: First, ATF3 was reported to play a role in both survival and proliferation of cancer cells [25,29-31]. Second, *ATF3 *transcript is expressed in ATL cells [27] although little is known about the biological significance of this expression, in particular whether expression of ATF3 is associated with ATL cell proliferation [27]. Third, the relation between ATF3 and HTLV-1 viral transcription is unknown. Immunoprecipitation analysis demonstrated that sHBZ and ATF3 interacted when transfected in mammalian cells (Figure 1). By using a series of truncated proteins, we found that bZIP domains of both sHBZ and ATF3 are necessary for their interaction.

### The ATF3 promoter is constitutively activated in ATL cell lines

**Figure 2:**
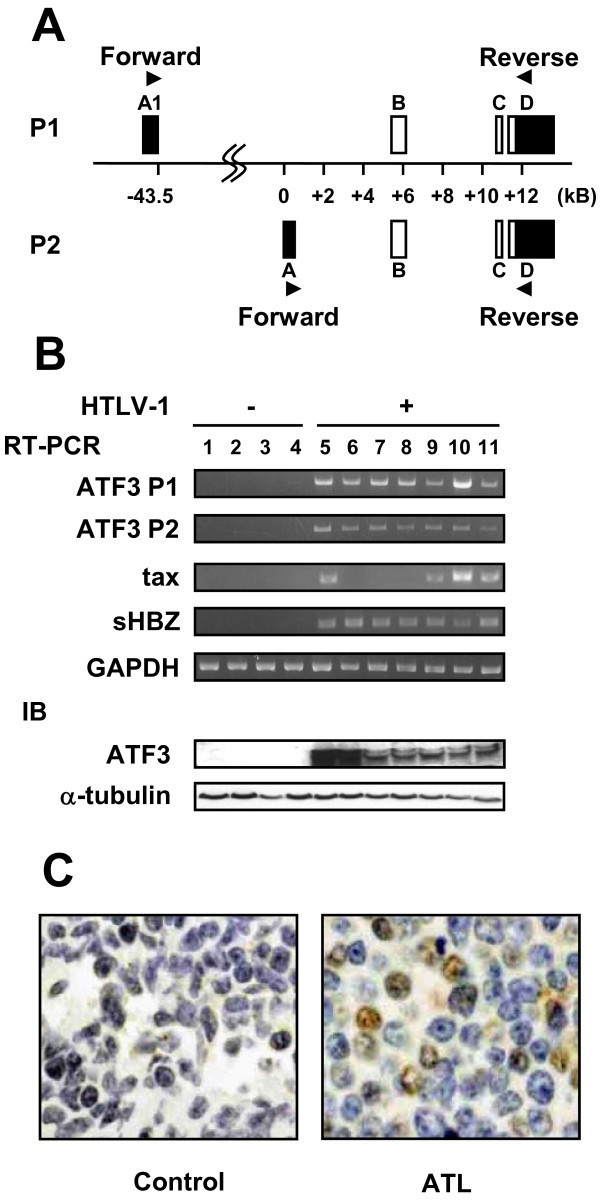
**Constitutive expression of ATF3 in ATL cells**. (A) Schematic diagram of the primers (arrowhead) for detecting transcripts from P1 and P2 promoters of the *ATF3 *gene. Square boxes represent the exons and white boxes represent open reading frame (ORF). Two distinct ATF3 transcripts that encode the same ORF are reported[32]. (B) *ATF3 *mRNA from P1 and P2 transcripts in HTLV-1 infected cell lines and ATL cell lines was determined by RT-PCR. Expression of ATF3 protein was studied by immunoblot (IB). Lane 1, Molt4; lane 2, CEM: lane 3. Kit225; lane 4, Jurkat; lane 5, ATL2; lane 6, ATL-43T; lane 7, ED; lane 8, TL-Om1; lane 9, MT-1; lane 10, MT-2; lane11, MT-4. (C) Immunostaining for ATF3 in lymph nodes of an ATL patient.

Next, we checked the expression level of *ATF3 *mRNA and protein in ATL cell lines. The *ATF3 *gene has two promoters: a non-canonical alternative promoter P1 and the canonical promoter P2 (Figure 2A) [32,33]. RT-PCR analysis demonstrated that all ATL cell lines constitutively expressed the *ATF3 *P1 and P2 transcripts (Figure 2B). ATF3 protein expression was also detected in all ATL cell lines (Figure 2B). In addition, all ATL cell lines expressed the s*HBZ *gene transcript while the *tax *gene was transcribed in only some ATL cell lines, consistent with earlier reports (Figure 2B) [6]. Although these data suggested that sHBZ expression might be associated with increased ATF3, ectopic expression of sHBZ did not induce *ATF3 *gene transcription in Jurkat cells (data not shown). Immunohistochemical analysis of lymph nodes of ATL patients showed that lymphoma cells indeed expressed ATF3 (Figure 2C).

### Suppressive effects of ATF3 on cellular and viral ATF/CRE sites

**Figure 3:**
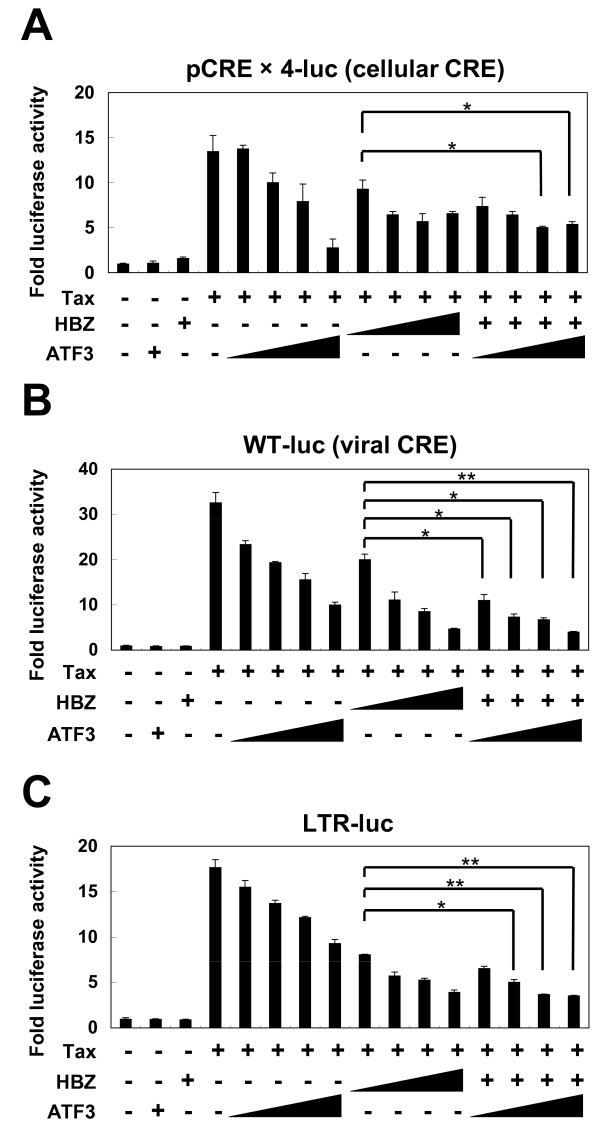
**Suppressive effects of ATF3 on Tax-mediated transactivation through ATF/CRE sites**. Jurkat cells were cotransfected with phRL-TK and expression vectors for ATF3, HBZ, and reporter plasmid pCRE × 4-luc (A), WT-luc (B), or LTR-luc (C) respectively. The total amount of DNA for transfection was equalized by adding empty vectors. After 24 hours, a dual luciferase reporter assay was performed as described in Materials and Methods. All the data are relative values of firefly luciferase normalized to Renilla luciferase and shown as a mean of a triplicate set of experiments (mean ± SD). *P <0.05; **P <0.01.

It has been reported that Tax activates the transcription of the plus strand of HTLV-1 as well as influencing host cellular gene transcription. Tax transcription of HTLV-1 genes depends on ATF/CRE-like sequences (viral CRE) in the U3 region of the HTLV-1 LTR [34,35]. ATF3, on the other hand, is reported to repress transcription from cellular ATF/CRE sites [36]. Based on these findings, we investigated whether ATF3 could influence Tax-mediated transcription. pCRE × 4-luc is a reporter construct containing a cellular ATF/CRE consensus sequence, while WT-luc contains ATF/CRE-like sequences from the HTLV-1 LTR. These plasmid DNAs were used to study the effect of ATF3 on transcription through cellular and viral CREs. Tax could activate the cellular and viral CRE reporters, but ATF3 by itself did not influence their activity (Figure 3A and 3B). ATF3 inhibited Tax-mediated transcription from ATF/CRE and viral CRE sites in a dose-dependent manner (Figure 3A and 3B). sHBZ also repressed Tax-mediated transcription, as reported previously [10]. When ATF3 and sHBZ were co-expressed, sHBZ did not inhibit the repressive function of ATF3. Next we checked the effect of ATF3 on Tax-mediated viral transcriptional activity. A reporter construct containing the entire HTLV-1 5'LTR was activated by Tax, as expected (Figure 3C). ATF3 repressed this transcription (Figure 3C). sHBZ also repressed Tax-mediated activation of this reporter, without interfering with the suppressive function of ATF3. These results suggest that ATF3 suppresses Tax-mediated ATF/CRE-dependent transcription both of cellular genes and the HTLV-1 LTR.

### ATF3 has growth promoting activity in ATL cells

**Figure 4:**
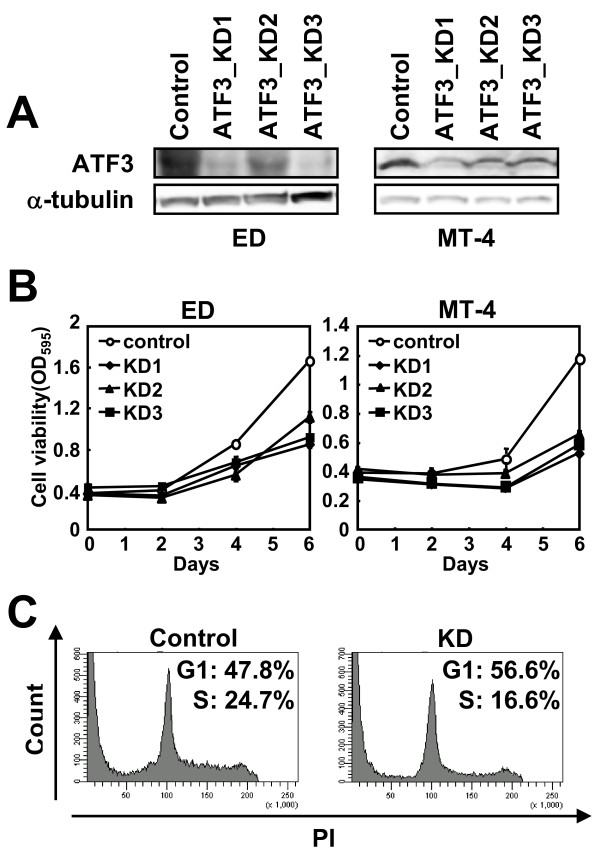
**Knockdown of ATF3 by shRNA impairs proliferation of ATL and HTLV-1 infected cells**. MT-4 and ED cells were transduced with lentivirus vector expressing control and ATF3-directed shRNA. (A) ATF3 protein was determined by immunoblot. (B) The cell growths of ATF3 knock-down ATL cells by shRNAs were measured by MTT assay. (C) The effect of ATF3 KD using ATF3_KD1 on cell cycle progression was analyzed by PI staining in MT-4 cells. Five days after infection, cells were analyzed by a flow cytometry as described in the Materials and Methods.

To investigate the functional significance of ATF3 expression in ATL cells, we transfected MT-4 and ED cells with lentiviral vectors expressing three different ATF3-directed shRNAs. These shRNA expressions strongly suppressed ATF3 protein expression shown in Figure 4A. An MTT assay showed that knockdown (KD) of ATF3 resulted in reduced proliferation of both Tax expressing MT-4 cells and Tax non-expressing ED compared to control cells (Figure 4B). Cell cycle analysis revealed that the population of G1 cells increased, while the population of cells in S phase decreased in ATF3 KD MT-4 cells (Figure 4C). KD of ATF3, then, impaired the G1/S transition in MT-4 cells, and hence ATF3 expression in ATL cell lines was associated with their proliferation.

### Transcriptional profile of ATF3 KD MT-4 cell

**Figure 5:**
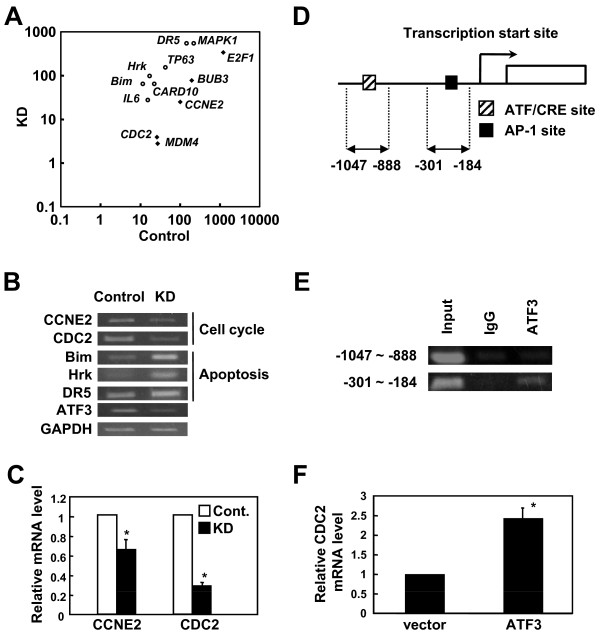
**CDC2 is a direct target of ATF3**. (A) The ratios of transcripts (Control/ATF3 KD populations) of 12 genes related to the cell cycle or apoptosis in the 2 groups are plotted. Open circles represent the up-regulated genes and black lozenges show the down-regulated genes. (B) The level of mRNA was studied by semi-quantitative RT-PCR to confirm the result of microarray analysis. (C) Control and ATF3 KD cells were analyzed by real-time PCR for the indicated mRNA. The expression level of control cells was defined as 1. Mean ± SD was based on results of three independent experiments (P < 0.01). (D) Schematic diagram of CDC2 primer used for ChIP assay. (E) 293FT cells were transfected with ATF3 expression vector. 48 hours after transfection, chromatin was prepared for a ChIP assay using an anti-ATF3 antibody. Anti-IgG was used as a negative control. (F) Jurkat cells were transiently transfected with ATF3 expression vector and CDC2 mRNA expression was measured by real-time PCR.

To find mechanisms by which ATF3 might increase proliferation, we performed oligonucleotide microarray analyses of ATF3-KD MT-4 cells and MT-4 cells transduced with a control vector. We compared the data from the negative control and ATF3-KD cells, and out of 18,400 transcripts, we first identified 2188 genes whose transcription changed more than two fold by KD. Of these, 1522 genes were up-regulated, and 658 down-regulated in ATF3-KD cells. Representative genes that were up-regulated or down-regulated by ATF3 are shown in Figure 5A and additional file 1.

We confirmed the expression of several up-regulated genes by RT-PCR to validate the results of the DNA microarray (Figure 5B). Suppressed expression of ATF3 increased the number of transcripts of proapoptotic genes, *Bim *and *Harakiri*. In contrast, cell division cycle 2 (CDC2) and cyclin E2 (CCNE2), which control the cell transition from G1 phase to S phase [37], were down-regulated in ATF3-KD cells. This is the first report that ATF3 affects the expression of these genes.

### CDC2 is a direct target of ATF3

Since KD of ATF3 impairs the G1/S transition, we focused on *cdc2 *and *ccne2 *gene expression. Quantitative analysis by real-time PCR confirmed that transcription of both the *cdc2 *and *ccne2 *genes was down-regulated in ATF3 KD cells compared to control cells (Figure 5C). The *cdc2 *gene expression was significantly decreased by KD of ATF3, so *cdc2 *gene was chosen for further studies. To study whether the effect of ATF3 on the *cdc2 *gene is direct or indirect, we investigated the binding of ATF3 to the promoter region of the *cdc2 *gene (Figure 5D). This region contains two putative binding sites for ATF3, an AP-1 site near the transcription start site, and an ATF/CRE site farther 5'-ward (Figure 5D). A chromatin immunoprecipitation assay detected ATF3 bound to the proximal AP-1 site, but ATF3 bound to ATF site was non-specific (Figure 5E). Transient transfection of Jurkat T cells by electroporation with a vector expressing ATF3 up-regulated the expression of *cdc2 *mRNA (Figure 5F). These results indicate that ATF3 directly activates transcription of the *cdc2 *gene.

### sHBZ inhibited the augmentation of p53 transcriptional activity by ATF3

**Figure 6:**
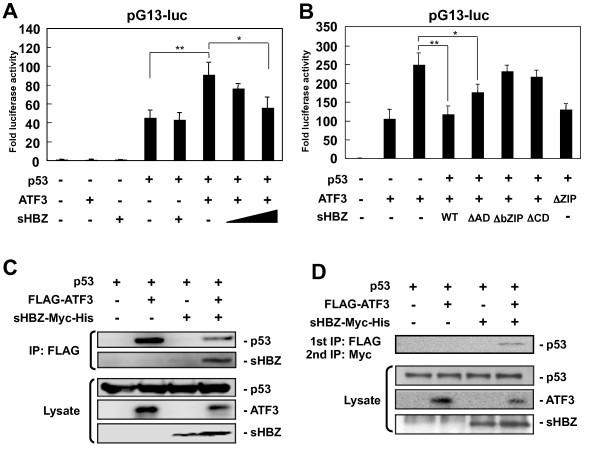
**HBZ inhibits the augmentation of p53 transcriptional activity by ATF3**. (A, B) Jurkat cells were cotransfected with phRL-TK and reporter plasmid pG13-luc and expression vectors for p53, ATF3 and HBZ or their deletion mutants. After 24 hours, a dual luciferase reporter assay was preformed. All the data shown are relative values of firefly luciferase normalized to Renilla luciferase and shown as the mean of a triplicate set of experiments (mean ± SD). *P <0.05; **P <0.01. (C, D) 293FT cells were transfected with p53, FLAG-ATF3, and sHBZ-Myc-His expression vectors. (C) Total cell lysates were subjected to IP using anti-FLAG followed by IB using anti-His and anti-p53. (D) Total cell lysates were subjected to a first IP step using anti-FLAG antibody. Immunocomplexes were eluted from anti-FLAG antibody-conjugated beads with FLAG peptide and then subjected to a second IP step using anti-Myc followed by IB using anti-p53.

In addition to its oncogenic function, ATF3 is also reported to augment transactivation of p53 responsive promoters in a non-small cell lung carcinoma cell line by protecting p53 from ubiquitin-associated degradation [31,38]. Expression of ATF3 in ATL cells therefore has the potential to promote apoptosis through p53, since mutations of p53 are rare in ATL cases [39]. To explore this possibility, we checked the ability of ATF3 to augment p53 transcriptional activity in T-cells. A reporter assay showed that, as reported previously [31,38], ATF3 enhanced transcriptional activity of p53 in ZIP domain dependent manner (Figure 6A and 6B). sHBZ, though it had no influence on p53 transcriptional activity itself, suppressed the increased transcriptional activity of p53 by ATF3 (Figure 6A). Analyses using sHBZ deletion mutants showed that the bZIP domain and the central domain of sHBZ were responsible for the suppressive activity (Figure 6B). To investigate how sHBZ reduces ATF3's ability to enhance p53 transcriptional activity, immunoprecipitation analyses were performed (Figure 6C). ATF3 interacted with p53 but sHBZ reduced this interaction. Serial immunoprecipitation experiments demonstrated that sHBZ, ATF3 and p53 were present in a complex together (Figure 6D). We propose that sHBZ binds directly to ATF3-p53 complexes; that this binding interferes, by unknown mechanisms, with ATF3 enhancement of p53 signaling; and that ATL cells expressing sHBZ can thereby escape the apoptosis that ATF3 expression might otherwise induce.

## Discussion

In this study, a yeast two-hybrid system identified ATF3 as a binding partner of the HTLV-1 sHBZ protein. Aberrant expression of ATF3 has been reported in classical Hodgkin lymphoma (cHL) and malignant prostate cancer cell [24,25], where it is associated with increased proliferation. In addition, increased expression of ATF3 was also reported in ATL cases [27]. However, the mechanism by which ATF3 promotes proliferation of cancer cells remained unknown. In this study, we demonstrated that increased expression of ATF3 was linked to proliferation via enhanced transcription of the *cdc2 *and *ccne2 *genes, along with suppressed expression of proapoptotic factors including Harakiri, and Bim. ATF3 indeed bound to the promoter region of the *cdc2 *gene and enhanced its transcription. Thus, ATF3 modulates transcription of cellular genes associated with proliferation and apoptosis.

ATF3 has been reported to act as transcriptional repressor of ATF/CRE sequences. In this study, we found that ATF3 suppressed activation, by the viral factor Tax, of transcription from CRE-like sequences in the 5'LTR. Tax, itself transcribed from the 5'LTR, is a major target of cytotoxic T-lymphocytes *in vivo *[40]. Therefore, suppression of *tax *gene transcription could benefit the survival of ATL cells, by allowing them to escape a cytotoxic T-lymphocyte response. In contrast to the *tax *gene, ATL cells need to express the *HBZ *gene transcripts for their proliferation [5]. HBZ is transcribed from the 3'LTR, and therefore unaffected by ATF3 suppression of the 5'LTR. By suppressing viral gene transcription through the 5'LTR, then, ATF3 modulates viral gene expression, favoring expression of the *HBZ *gene over the *tax *gene. Enforced expression of ATF3 in prostate cancer cells induces cell proliferation and accelerates progression from the G1- to S-phase of the cell cycle [25]. The same study also showed that KD of ATF3 expression decreased cells in S phase while it increased cells in G1 phase [25]. In addition, impaired G1/S transition in c-myc null cells was partially recovered by ATF3 expression [30], indicating the role of ATF3 in G1/S transition.

In this report, we present evidence that the expression of ATF3 is associated with G1/S progression via enhanced transcription of the *cdc2 *and *ccne2 *genes, and possibly others. In particular, ATF3 bound the CDC2 promoter directly. The *cdc2 *gene plays a key role in the transition from the G1 phase to the S phase [41], and from the G2 phase to the M phase. The *ccne2 *gene is reported to be highly expressed in a number of human primary tumors including breast, ovary, uterus, brain, and lung [42]. Our results now open the possibility that *ccne2*, as well as *cdc2*, may contribute to ATL as well.

Independent of its cell cycle-promoting function, ATF3 also acts like a tumor suppressor, enhancing p53 transcriptional activity by inhibiting its ubiquitin-mediated degradation [31,38]. ATF3 neither interferes with the p53-MDM2 interaction nor blocks the E3 ligase activity of MDM2, suggesting that binding of ATF3 to p53 likely induces a conformational change of p53 that inhibits ubiquitination [31,38]. Since *ATF3 *is an adaptive response gene that responds to extra or intracellular changes, ATF3 stabilization of p53 counters cellular stress due to environmental insult and ensures genomic integrity [31,38]. Given that *p53 *is mutated in only about 30% of ATL cases [43-45], and in fact the expression level of p53 protein increases in ATL cells [46], how is ATF3's p53-stabilizing activity consistent with the chromosome instability often observed in ATL cells [47]? In fact, post-translational inactivation of p53 is critical to understanding ATL development. A viral protein, Tax, can functionally inactivate p53 by competing for binding to E-box [48], as well as other mechanisms [49]. However, Tax is not expressed in many ATL cases, due to genetic and epigenetic changes of the HTLV-1 provirus [5,50], including nonsense mutations generated by APOBEC3G [51]. Mechanisms other than Tax must therefore interfere with p53 signaling. As shown in this study, sHBZ binds to ATF3-p53 complexes. With these interactions, sHBZ reduces ATF3's ability to enhance p53 function. HTLV-1 is not unique in deploying viral proteins to perturb p53 function. The latency-associated nuclear antigen encoded by Kaposi's sarcoma-associated herpesvirus, for example, binds to von Hippel-Lindau factor and targets it for degradation[52]. The human papilloma virus-encoded E6 protein binds to the cellular E6-associated protein (E6AP), an ubiquitin ligase that targets p53 for destruction. In fact, this interaction is blocked by ATF3, revealing another way in which ATF3 reinforces p53 signaling [53].

In HTLV-1's case, sHBZ perturbs one ATF3 function - p53 stabilization - that might slow the proliferation of infected cells, while leaving other functions - promotion of G1/S transition, and repression of provirus transcription - unaffected. HTLV-1 reproduces mainly by promoting the clonal expansion of infected cells, rather than by producing new virus particles. As such, the potential benefits to the virus of modulating ATF3 function in this way are clear: ATF3, in combination with sHBZ, encourages infected cells to progress through the G1/S phase transition, unimpeded by a ATF3-p53 response, and free from detection by host immune cells that might recognize viral antigens transcribed from the 5'LTR.

## Conclusions

This study reveals a role of ATF3 in regard to proliferation and viral gene transcription in ATL cells. The combined effects of ATF3 and sHBZ allow ATL cells to survive *in vivo*, and could be a target of therapy for this malignant disease.

## Methods

### Cell lines

All T-cell lines and ATL cell lines were grown in RPMI 1640 supplemented with 10% fetal bovine serum and antibiotics. 293FT cells were cultured in Dulbecco modified Eagle medium supplemented with 10% FBS and 500 μg/ml G418.

### Yeast two-hybrid

A yeast two-hybrid screen was performed by Hybrigenics (http://www.hybrigenics.com) on a random-primed Leukocytes and Activated Mononuclear Cells cDNA library using HBZ as bait.

### Plasmids

The ATF3 coding sequence was amplified by polymerase chain reaction (PCR) and was cloned into pCMV-Tag2 (Stratagene, La Jolla, CA), or pcDNA3 (Invitrogen, Carlsbad, CA). Expression vectors for sHBZ [28], its deletion mutants [28], reporter plasmids pWT-luc, pLTR-luc [34,35], and pG13-luc [54] were described previously. pCREx4-luc was purchased from Stratagene (La Jolla, CA). Luciferase assay was performed as described previously [12].

### Knockdown analysis

Cells were infected with an shRNA lentiviral vector (Invitrogen) directed against ATF3. The following target sequence were chosen: ATF3_KD1 5'-GAGCTGAGGTTTGCCATCC-3', ATF3_KD2 5'-GTGTATTGTCCGGGCTCAG-3' and ATF3_KD3 5'-GAACGAGAAGCAGCATTTG-3' as described previously [24]. Control cells were infected with an shRNA retroviral vector expressing a nonsilencing construct provided also by Invitrogen that does not target any known vertebrate gene as described in manufacture's instruction.

### Proliferation assay and cell cycle analysis

Cell viability was measured with a 3-(4,5-dimethylthiazol-2-yl)-2,5-diphenyltetrazolium bromide (MTT) colorimetric assay [55]. In cell cycle analysis, after cell fixation with 70% ethanol, cells were suspended in 50 μg/ml Propidium Iodide solution containing 0.1 mg/ml RNase A and 0.05% Triton X-100 for 40 min at 37℃ and were analyzed by flow cytometry.

### Immunohistochemical analyses

The tissue specimens were obtained from human lymph nodes filed at the Department of Pathology at Kurume University. The study of clinical samples was approved by the local research ethics committee of the Kurume University. Tissue samples were fixed in 10% formalin in phosphate buffer and then embedded in paraffin and analyzed by immunohistochemical methods to determine ATF3 expression. Images were captured using a Provis AX80 microscope equipped with an OLYMPUS DP70 digital camera, and detected using a DP manager system (Olympus, Tokyo, Japan).

### Electroporation

Electroporation was performed with Neon™ transfection system (Invitrogen). Electroporation parameters for Jurkat cell were those recommended by Invitrogen.

### RNA isolation, Reverse transcriptase (RT)-PCR, real-time PCR

Total RNAs were extracted using TRIZOL (Invitrogen) according to the manufacturer's protocol. Primers for the *ATF3*, *HBZ*, and *tax *genes were described previously[6,32] The Power SYBR Green PCR Master Mix (Qiagen, Venlo, Netherlands) was used in real-time PCR analysis in triplicate with β-actin as an internal control. In general, the threshold cycle numbers for actin in different cells are very close, and the relative mRNA level for the gene of interest is calculated as 2^[Ct (actin)-Ct (gene)]^, where Ct is threshold cycle number. Primers were 5'-TGGAAACCAGGAAGCCTAGC-3' (sense) and 5'-GAAATTCGTTTGGCTGGATCAT-3' (antisense) for CDC2; 5'-GAATGTCAAGACGAAGTA-3' (sense) and 5'-ATGAACATATCTGCTCTC-3' (antisense) for CCNE2.

### Oligonucleotide microarray analysis

RNA processing and hybridization to U133 Plus 2.0 GeneChip microarrays were performed according to the manufacturer's protocol (Affimetrix, Santa Clara, CA). Data were analyzed with the GeneSpring GX 10 software (Agilent Technologies, Palo Alto, CA).

### Immunoprecipitation (IP) and immunoblotting

Cell lysates were incubated with anti-His-Tag (PM002) (MBL, Nagoya, Japan), anti-c-myc (clone 9E10) and anti-FLAG M2 antibodies (Sigma-Aldrich, St Louis, MO) for 1 hour at 4°C, and immune complexes were incubated with protein G-sepharose (GE Healthcare, Little Chalfont, UK) for 1 hour at 4°C. The following antibodies were used for immunoblot: anti-ATF3 (Santa Cruz Biotechnologies, Santa Cruz, CA); anti-His-Tag (PM002) (MBL); anti-FLAG M2 and anti-p53-biotin (Sigma-Aldrich); peroxidase-conjugated anti-mouse IgG or anti-rabbit IgG or streptavidin-biotinylated horseradish peroxidase complex (GE Healthcare). To detect ATF3 using anti-ATF3 antibody, Immuno-enhancer (Wako, Osaka, Japan) was used.

### Serial IP

Cells were lysed in lysis buffer (50 mM Tris-HCl [pH 7.5], 150 mM NaCl, 1 mM EDTA, 0.5% NP-40, and protease inhibitor cocktail), and incubated for 1 hour at 4°C. For the first IP, after clarification by low-speed centrifugation, the supernatants were incubated with anti-FLAG M2 Affinity gel (Sigma-Aldrich) for 3 h at 4°C. The FLAG-agarose beads were then washed with lysis buffer and the bound proteins were eluted with FLAG elution buffer (50 mM Tris-HCl, 150 mM NaCl, and 0.5 mg/ml FLAG peptide [Sigma]) for 1 hr at 4°C. For the second IP, after the FLAG-agarose beads were removed by centrifugation, the supernatants were incubated with anti-c-Myc for 1 hr at 4°C.

## Competing interests

The authors declare that they have no competing interests.

## Authors' contributions

This study was designed by KH and MM. Laboratory analysis was performed by KH. Data analysis was performed by KH, SY, YJ and MM. Samples and data were provided by OK. KH and MM wrote the paper. All authors read and approved the final manuscript.

## Supplementary Material

Additional file 1 **Figure S1**. Identification of candidate genes regulated by ATF3 expression. Oligonucleotide microarray data for control and ATF3 KD MT-4 cells were subjected to cluster analysis with the GeneSpring GX 10 software. Each column represents expression level of a given gene. Red represents increased expression and green represents decreased expression relative to the normalized expression of the gene across all samples. Click here for file
