# Supplementary material for: ATF3, an HTLV-1 bZip factor binding protein, promotes proliferation of adult T-cell leukemia cells
Source: Retrovirology. 2011 Mar 17;8:19. doi: 10.1186/1742-4690-8-19 (PMC3068935; doi:10.1186/1742-4690-8-19)
Supplement: Additional file 1 — Figure S1. Identification of candidate genes regulated by ATF3 expression. Oligonucleotide microarray data for control and ATF3 KD MT-4 cells were subjected to cluster analysis with the GeneSpring GX 10 software. Each column represents expression level of a given gene. Red represents increased expression and green represents decreased expression relative to the normalized expression of the gene across all samples. [file 1742-4690-8-19-S1.PPT]

## Slide 1
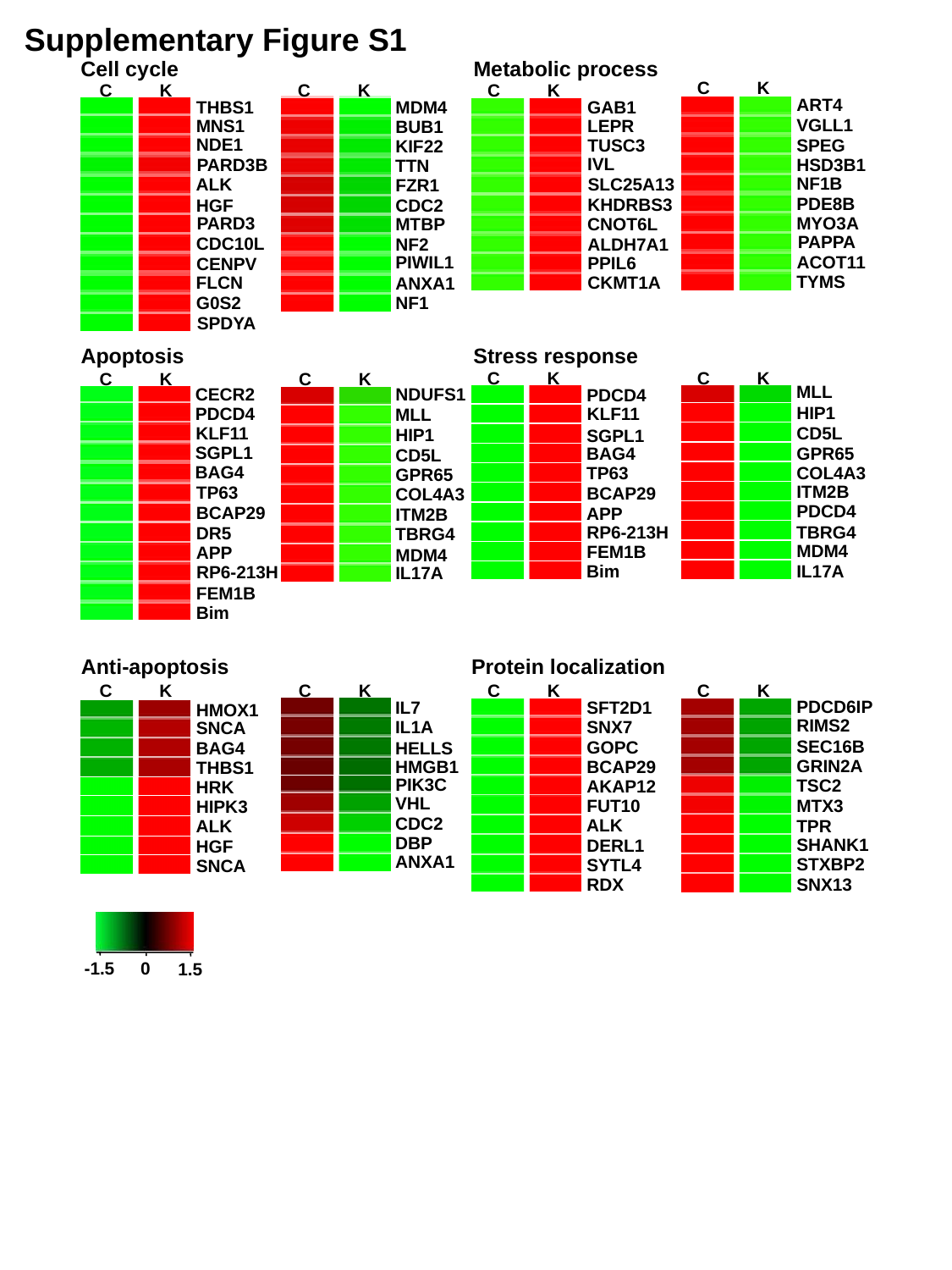

Supplementary Figure S1
Cell cycle
C
K
C
K
THBS1
MDM4
MNS1
BUB1
NDE1
KIF22
PARD3B
TTN
ALK
FZR1
HGF
CDC2
PARD3
MTBP
CDC10L
NF2
PIWIL1
CENPV
FLCN
ANXA1
G0S2
NF1
SPDYA
Metabolic process
C
K
C
K
ART4
GAB1
VGLL1
LEPR
SPEG
TUSC3
IVL
HSD3B1
NF1B
SLC25A13
PDE8B
KHDRBS3
MYO3A
CNOT6L
PAPPA
ALDH7A1
ACOT11
PPIL6
TYMS
CKMT1A
Apoptosis
C
K
C
K
CECR2
NDUFS1
PDCD4
MLL
KLF11
HIP1
SGPL1
CD5L
BAG4
GPR65
TP63
COL4A3
BCAP29
ITM2B
DR5
TBRG4
APP
MDM4
RP6-213H
IL17A
FEM1B
Bim
Stress response
C
K
C
K
MLL
PDCD4
HIP1
KLF11
CD5L
SGPL1
GPR65
BAG4
TP63
COL4A3
ITM2B
BCAP29
PDCD4
APP
RP6-213H
TBRG4
MDM4
FEM1B
Bim
IL17A
Anti-apoptosis
C
K
C
K
IL7
HMOX1
IL1A
SNCA
BAG4
HELLS
HMGB1
THBS1
PIK3C
HRK
VHL
HIPK3
CDC2
ALK
DBP
HGF
ANXA1
SNCA
Protein localization
C
K
C
K
PDCD6IP
SFT2D1
RIMS2
SNX7
SEC16B
GOPC
GRIN2A
BCAP29
TSC2
AKAP12
FUT10
MTX3
ALK
TPR
SHANK1
DERL1
STXBP2
SYTL4
RDX
SNX13
-1.5
0
1.5
